# An Elderly Lady with Fever of Unknown Etiology and Severe Pulmonary Hypertension: Intravascular Lymphoma—An Elusive Diagnosis

**DOI:** 10.1155/2013/153798

**Published:** 2013-12-19

**Authors:** Nupur Sinha, Luis Lantigua, Masooma Niazi, Gilda Diaz-Fuentes

**Affiliations:** ^1^Division of Pulmonary and Critical Care Medicine, Bronx Lebanon Hospital Center, Affiliated with Albert Einstein College of Medicine, Bronx, NY 10457, USA; ^2^Department of Internal Medicine, Bronx Lebanon Hospital Center, Affiliated with Albert Einstein College of Medicine, Bronx, NY 10457, USA; ^3^Department of Pathology, Bronx Lebanon Hospital Center, Affiliated with Albert Einstein College of Medicine, Bronx, NY 10457, USA

## Abstract

Pulmonary hypertension (PH) associated with malignancy, especially adenocarcinoma, is a well-known entity and is included in group V of the WHO classification. Intravascular lymphoma is a rare type of diffuse large B cell lymphoma, characterized by selective intravascular growth of malignant lymphocytes, aggressive behavior, and often a fatal course. Most of the time, diagnosis is postmortem due to the rarity and the protean manifestations of the disease. We present a rare case of an elderly patient presenting with severe pulmonary hypertension, fever of unknown etiology (FUO), and lymphadenopathy. Extensive evaluation searching for the etiology of her FUO and PH was noncontributory. The diagnosis of intravascular lymphoma was finally reached by the performance of a random abdominal fat pad biopsy and the patient was started on immunochemotherapy. She continues the follow up after 6 cycles of R-CHOP with no further febrile episodes and steady improvement in exercise tolerance.

## 1. Introduction

The prevalence of all types of pulmonary hypertension (PH) is not well defined; it is estimated that 10%–20% of the general population has PH by echocardiography [1]. Group 1 PH is the most widely studied and accounts for approximately 15 cases per million, but it is the other etiologies of PH which are more common. While left heart failure accounts for >65% of all clinically detected PH, the incidence and prevalence of other groups, especially group V, are less clearly defined [1]. The 5-year mortality across all groups is comparable and is estimated to be approximately 34% [1].

Fever of unknown etiology (FUO) is one of the most challenging clinical presentations for the clinician. It may be associated with infections, malignancy, drug or toxin intake, and environmental toxicity among others. In many cases, despite extensive investigation, the etiology remains unclear [2].

In a patient presenting with FUO and PH, the main differential diagnoses focus on collagen vascular diseases (CVD) or vasculitis, sarcoidosis, and malignancy. PH associated with malignancy is a well-known entity and it is included in group V of the WHO classification of PH. The two most commonly proposed mechanisms for PH in this group are pulmonary tumor thrombotic microangiopathy (PTTM) and pulmonary tumor embolism (PTE). Adenocarcinoma, mainly of gastrointestinal etiology, is the most common malignancy associated with PTTM and related to severe PH [3–5].

We present a rare case of intravascular lymphoma (IVL) presenting with FUO, severe PH, and disseminated lymphadenopathy diagnosed by random fat pad biopsy.

## 2. Case Presentation

A 72-year-old Hispanic woman from the Dominican Republic presented with unexplained intermittent fever of several months duration. She had multiple hospital admissions for similar complaints. Her medical history included diabetes mellitus, hypertension, dyslipidemia, end-stage renal disease, chronic anemia, atrial fibrillation (not on anticoagulation), history of cardiac arrest, and diastolic heart failure. Surgical history included C-section, umbilical herniorrhaphy, arteriovenous fistula, and appendectomy.

She denied any recreational habits or occupational exposure. She traveled to her country almost every year. Family history was not significant. Purified protein derivative (PPD) was negative 5 years ago and age appropriate screenings (mammogram and colonoscopy) were normal. She reported intermittent fever with chills, myalgias, fatigue and weight loss of 20 pounds in 6 months. In addition, she had vague abdominal discomfort and progressive dyspnea on exertion with decreased exercise tolerance to less than 1 block. She denied any cough, joint or chest pain, palpitations, rash, any bleeding, or other gastrointestinal complaints. Her physical exam was significant for pallor, pedal edema, mild axillary adenopathy, systolic ejection murmur, ascites, and non-tender hepatomegaly. There were no new findings compared with prior examination several months prior to this evaluation.

Laboratory parameters revealed chronic pancytopenia, chronic kidney failure, and elevated lactate dehydrogenase (LDH) at 500 units/L. LDH had ranged from 216 to 602 in the past 6 months. Electrocardiography showed an old right bundle branch block with right axis deviation.

Prior investigation for FUO included the following.

**Figure 1:**
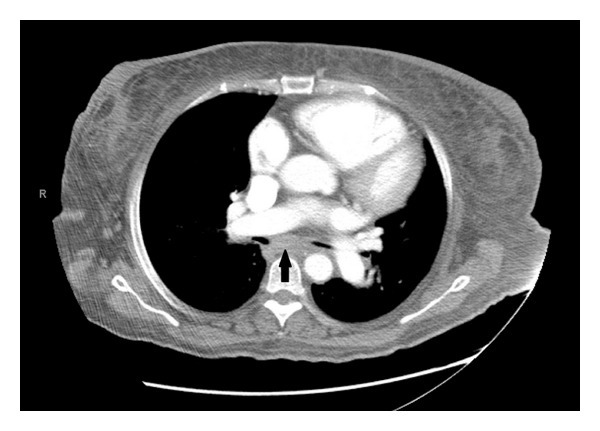
Chest CT showing mediastinal lymphadenopathy (arrow).

- Septic workup including aerobic, acid fast bacilli, viral and fungal cultures, studies for histoplasma, legionella, mycoplasma, ehrlichia, babesia, human immunodeficiency virus, rickettsia, rocky mountain spotted fever, cytomegalovirus, and malaria were all negative. Stool blastomyces was positive and treated.
- Hepatitis profile was negative. Epstein Barr virus IgM was elevated at 1.9. Parvovirus IgG was high at 4.8 and HTLV-1 was negative.
- Ophthalmology and otorhinolaryngology exams were noncontributory.
- MRI of spine, Gallium scan, and head CT were normal.
- Chest X-ray revealed cardiomegaly with prominent pulmonary artery. Chest/abdomen/pelvic CT revealed mediastinal and para-aortic adenopathy, hepatomegaly, and ascites. Due to the presence of multiple adenopathies and elevated angiotensin converting enzyme with levels of 189 U/L, the patient was evaluated for sarcoidosis versus a lymphoproliferative disorder. Fiberoptic bronchoscopy with bronchoalveolar lavage and subcarinal lymph node (LN) (Figure 1) biopsy was negative for granulomatous or neoplastic disorder.
- Transthoracic echocardiogram a few months ago showed severe PH with RVSP 60 mmHg with estimated EF 60% and transesophageal echocardiogram was negative for infection.
- Bone marrow biopsy revealed normocellular marrow with erythroid predominance and trilineage mutation; left axillary LN biopsy was reported as reactive adenopathy.
- Vasculitis workup had shown positive ANA (1 : 320) titer with polyclonal gammopathy in serum protein electrophoresis. Rheumatoid factor, anti-Sm, anti-RNP, and C4 were normal. Anti-DNA Ab was mildly elevated at 10, and C3 was low at 84. Serum immunofixation was negative. The patient was given a presumptive diagnosis of lupus on a prior admission and had received steroids and nonsteroidal anti-inflammatory intermittently with no improvement in condition.

During the present admission, an abdominal paracentesis was negative for infection or malignancy and was consistent with noncirrhotic etiology. ADA level in ascitic fluid was 15.9 IU/L (cut-off value of 39 IU/L) and serum quantiferon was indeterminate.

As the clinical presentation was not suggestive of lupus, in absence of rash/hypersensitivity, arthralgias, oral ulcers, or pleuropericardial involvement and normal repeated labs including DNA antibody, antihistone Ab, anti-Sm antibody, rheumatoid factor as well as anti-CCP, it was concluded by rheumatology that it was very unlikely for the patient to have lupus.

A ventilation-perfusion (V/Q) scan showed low probability for pulmonary embolism. Repeated transthoracic echocardiogram showed persistent severe PH (RVSP 89 mmHg, normal LV EF 58% with abnormal septal motion consistent with right ventricular volume, and pressure overload with severe TR). RVSP had worsened significantly compared to the prior echocardiogram with the rest of the echocardiographic parameters remaining unchanged. EKG showed LVH with low voltage. An abdominal fat pad biopsy was performed to evaluate for possible amyloidosis in view of chronic renal failure, cardiac findings, and FUO.

**Figure 2:**
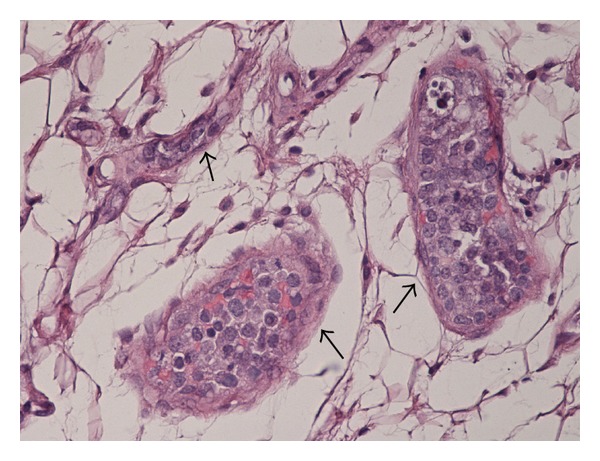
Histopathology of fat pad biopsy showing intravascular large B cell lymphoma cells mainly located in lumina of small vessels (arrows).

**Figure 3:**
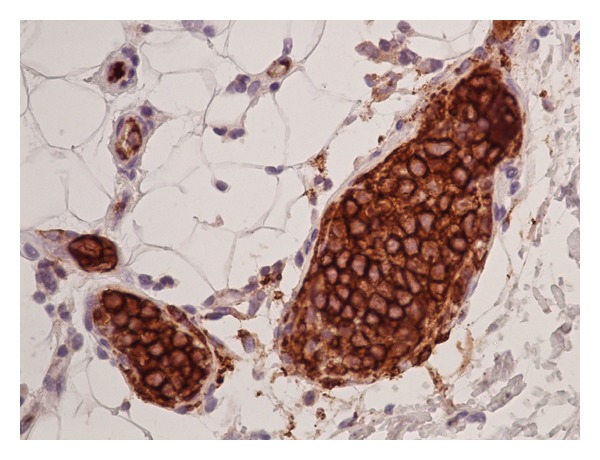
Histopathology of fat pad biopsy showing the intravascular large B cell lymphoma cells immunoreactive to CD20.

Histopathology of the abdominal fat pad showed adipose tissue with numerous small blood vessels, predominantly capillaries and venules (Figure 2). Lumens filled with large lymphoma cells were immunoreactive to B cell markers CD20 and CD79a (Figure 3), and negative for T cell markers.

A diagnosis of intravascular B cell lymphoma with B symptoms was made and the patient was started on immunochemotherapy. She received adjusted dose R-CHOP X6, followed by Rituxan X2. Although repeated echocardiogram 6 months after initiation of chemotherapy reveals no significant change from the previous one, she has demonstrated significant clinical improvement in appetite, alertness, and effort tolerance. Initially classified as WHO functional class IV at the time of diagnosis, after 6 months of follow up, she is now at WHO class III. Blood flow cytometry performed at the initiation of immunochemotherapy showed circulating B cell lymphoma expressing CD19, CD20, and CD23. Repeated cytometry after 6 months of immunochemotherapy shows no lymphoma cells. Patient has no further febrile episodes and LDH is normalized to 182 units/L.

## 3. Discussion

Intravascular lymphoma (IVL) is a rare type of diffuse large B cell lymphoma (DLBCL), with an estimated incidence of less than one person per million, and is characterized by selective intravascular growth of malignant lymphocytes, aggressive behavior, and often a fatal course [6]. Initially described by Pflegrand and Tappeiner in 1959 as “angioendotheliomatosis proliferans systemisata” and in 2008 defined by the World Health Organization (WHO) as intravascular large B cell lymphomas (IVBCL), IVL is a type of extra-nodal large B cell lymphoma where growth is restricted to the lumina of the vessels, particularly the capillaries [7]. It is characterized by an extremely heterogeneous presentation with involvement of small vessels of nearly every organ. IVBCL is considered to be a disseminated disease at the time of diagnosis, which is often postmortem due to the protean presentation and the rarity of the entity. IVBCL has been described in adult patients of all ages with a median of 70 years. It occurs equally in women and men. There are no known risk factors for IVL; however, there are reports of IVL arising in the setting of a diagnosis of DLBCL, follicular lymphoma and treated solid tumors. Median reported survival is 12 months [6–8].

The largest study of IVL by Ferreri and collaborators, described fever as the most commonly presented symptom. The brain and skin were the most frequently involved organs, with 68% of the patients having involvement of at least one of these organs. Involvement of bone marrow was found in one-third of the patients with IVL and was associated with hepatosplenic involvement and pancytopenia. In 11% of cases, LN was involved [9, 10]. A distinct “Asian variant” is recognized in Japanese patients with hemophagocytic disorder [11]. Additionally, there are isolated reports of IVL affecting almost every organ system such as interstitial lung disease, adrenal failure, pulmonary hypertension, nephrotic syndrome, myocardial infarction, and symmetric polyarthritis. Although IVL is a clonal proliferation of lymphocytes, extranodal distribution is the hallmark, and it is uncommon to find significant adenopathy, hepatosplenomegaly, or circulating cells in the peripheral blood.

An increased serum LDH and b2-microglobulin are found in more than 80% of the cases, anemia in two-thirds, elevated erythrocyte sedimentation rate in 43%, and monoclonal serum component in 14% of the cases [11]. Abnormal hepatic, renal, and endocrine functions and increased CSF protein levels are common in IVL and are associated with organ involvement by lymphoma cells [6–10, 12].

The diagnosis is often delayed due to varied clinical presentations and limited understanding of the entity. The knowledge of IVL is limited to isolated reports and cumulative reviews. IVL is potentially a systemic disease and diagnosis by random skin, muscle, and fat pad biopsy has been described [13, 14]. Histopathology remains the gold standard for diagnosis, showing the classic appearance of large malignant lymphocytes filling small vascular lumina. The affected vessels are usually small to medium sized structures. The neoplastic cells are usually large, with high nuclear-cytoplasmic ratio and scant cytoplasm. The classic immunophenotype of the malignant lymphocyte in IVL is B cell-associated antigen-positive (CD19+CD20+CD22+CD79a+). Several cases have been reported to express CD5. The B-cell phenotype is described in 91% and the T cell phenotype in 9% of IVL cases. Two cases of the natural killer cell phenotype have also been reported [6–10, 15].

Treatment recommendations are extrapolated from results of trials of more common subtypes of lymphoma. Most cases of IVL are associated with poor prognosis and should be treated systemically with an anthracycline-based regimen. Anthracycline-based chemotherapy has been associated with a 60% response rate and a 3-year overall survival rate of more than 30%. CHOP and CHOP-like regimes are considered to be also effective [16]. The introduction of rituximab has led to the doubling of progression-free survival and overall survival [17].

Consistent with other reports, there was substantial delay in reaching diagnosis in our patient. The severe PH with rapid deterioration in our patient could not be explained only by stable diastolic heart failure and ESRD. Pulmonary IVL has been described and is characterized by multifocal disease leading to dyspnea, air trapping, and severe PH with rapidly aggressive behavior [18]. While lung involvement has been demonstrated at autopsy in approximately 60% of the cases, pulmonary symptoms are distinctly unusual at presentation. Pulmonary hypertension associated with IVL has been rarely described. Review of the English literature revealed only four cases of IVL and PH or suspicion of pulmonary embolism with only one of these cases diagnosed while alive. The mechanism of the PH as suggested by autopsy findings is occlusion of the lumina of pulmonary arteries, venules, and capillaries by intraluminal malignant cells and segmental angiitis [19–21]. In our patient, extensive workup ruled out infectious causes including HIV, paracentesis and imaging ruled out portal hypertension, hematology excluded hemoglobinopathy or chronic hemolytic anemia, and repeat rheumatology workup ruled out any connective tissue disease. There was no evidence of sarcoidosis on FOB-TBNA and axillary LN biopsy. Repeated admissions and labs had not demonstrated any evidence of hypoxia or hypercarbia in a nonsmoker patient. There were no bases to suspect sleep apnea and imaging did not reveal any underlying interstitial disease. Preserved and stable left ventricular function on echocardiogram made a postcapillary PH contributing to rapid deterioration less likely and investigations did not show thromboembolic disease. While absence of right heart catheterization was a big limitation and we do acknowledge that diastolic heart failure was the most likely underlying cause of PH in our patient to begin with, we postulate that widespread obliteration of pulmonary vasculature by the lymphoma is the most likely explanation for the rapid deterioration of PH in our patient. Although no significant echocardiographic response could be demonstrated at 6-month follow up, it is noteworthy that the link between PH and IVL has been described mainly in autopsy reports. Georgin-Lavialle et al. [22], reported a single case diagnosed antemortem, where IVL mimicking PE was described. They had noted an association with PH but no mention of echocardiographic findings was made and the case was followed clinically. While multiple studies have been performed on group I PH and the response to experimental drugs has been predominantly measured by clinical outcome and comparison of effort tolerance, the response to treatment in other groups is not clear in the literature [23, 24]. In addition, as noted in earlier studies, it may be difficult to obtain accurate pulmonary capillary wedge pressure in the presence of intrinsic pulmonary vascular disease [25], indicating low probability of reliable assessment of pathological response in our case. Her clinical response and improvement from WHO functional class IV to class III after immunochemotherapy favor our hypothesis linking the PH with IVL.

## 4. Conclusion

Undiagnosed intravascular lymphoma carries a high mortality, and the diagnosis remains a challenge that requires a very high index of suspicion. This entity can affect any organ of the body, and due to the diverse presentation, these patients are usually evaluated by multiple specialties. IVL should be strongly considered in patients with FUO and PH, especially in the presence of elevated serum LDH. We recommend a low threshold for random skin, muscle, or fat pad biopsy in patients with unexplained or out of proportion pulmonary hypertension.

## Abbreviations

CT: Computed tomography
CVD: Collagen vascular disease
DLBCL: Diffuse large B cell lymphoma
EF: Ejection fraction
FUO: Fever of unknown origin
IVBCL: Intravascular B cell lymphoma
IVL: Intravascular lymphoma
LDH: Lactate dehydrogenase
LN: Lymph node
LVH: Left ventricular hypertrophy
MRI: Magnetic resonance imaging
PH: Pulmonary hypertension
PTE: Pulmonary tumor embolism
PTTM: Pulmonary tumor thrombotic microangiopathy.
